# Differences in avidity of anti-post-translationally modified protein antibodies in mouse models and rheumatoid arthritis patients: not one-size-fits-all

**DOI:** 10.1136/rmdopen-2024-004131

**Published:** 2024-05-24

**Authors:** Stef van der Meulen, Lu Zhang, Jacqueline M F van Veenendaal, Diane van der Woude, Leendert A Trouw

**Affiliations:** 1 Department of Immunology, Leiden University Medical Center, Leiden, The Netherlands; 2 Department of Rheumatology, Leiden University Medical Center, Leiden, The Netherlands

**Keywords:** Autoantibodies, Anti-Citrullinated Protein Antibodies, Arthritis, Rheumatoid

Autoantibodies against post-translationally modified (PTM) proteins exist in several autoimmune diseases. In rheumatoid arthritis (RA), anti-citrullinated protein antibodies (ACPAs) are part of the classification criteria, and both ACPA and anti-carbamylated protein (CarP) antibodies provide prognostic information.^1^ Studies into the ACPA and anti-CarP antibody responses offer insight into disease onset and progression.

Cumulative data indicate that the B-cell response producing ACPA matures, as supported by rising autoantibody levels, isotype switching and epitope spreading.^2^ However, interestingly, we, and recently Yamada *et al*,^3^ showed that the avidity of ACPA is low compared with recall antigens in the same patients.^4^ While Yamada *et al* compared the ACPA avidity to other autoantibody responses and showed that ACPA really stands out with its low avidity compared with other autoantibodies in RA, we wondered whether the ACPA response would be different from other anti-PTM responses. We previously studied the avidity of anti-CarP antibodies in RA and observed that the anti-CarP response is of low avidity.^5^ Here, we compare the avidity of different anti-PTM responses using mouse experiments and sera of RA patients.

First, we studied anti-PTM antibody response and avidity in a mouse model. Six different PTMs were studied: citrullination (Cit), carbamylation (CarP), acetylation (AL), malondialdehyde-acetaldehyde adducts (MAA), nitration (NT) and advanced glycation end-products (AGE). These PTMs were generated on mouse serum albumin (MSA), which is a mouse self-protein. We used the unmodified MSA as a negative control and the non-self protein ovalbumin as a positive control. The methods are described in online supplemental information.

10.1136/rmdopen-2024-004131.supp1 Supplementary data

**Figure 1:**
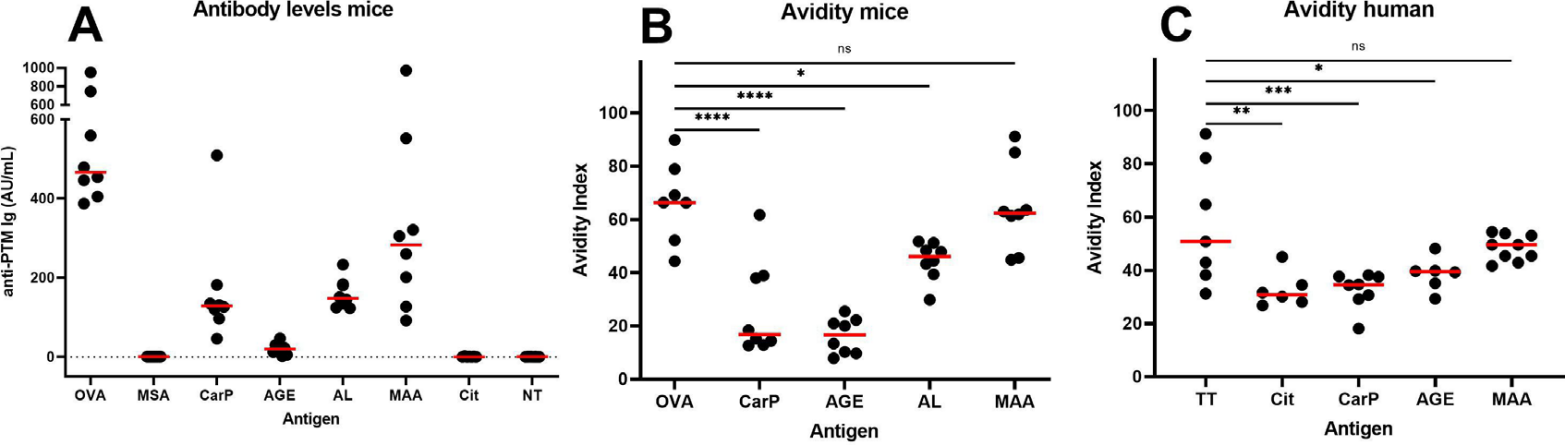
(A) Specific antibody levels of immunised mice in different groups (n=8 in all groups). We observe an antibody response in the CarP, AGE, AL and MAA groups. OVA was used as positive control. (B) Avidity index of the mice that had an antibody response (OVA n=7. All other groups n=8). Avidity of CarP, AGE and AL is lower compared with the ovalbumin control. (C) Avidity index of human samples where we observed low avidity antibodies for Cit, CarP and AGE compared with the control tetanus toxoid (TT) (TT n=7, Cit n=6, CarP n=8, AGE n=6 and MAA n=9). Statistics: ns p>0.05, *p≤0.05, **p≤0.01, ***p≤0.001, ****p≤0.0001. AGE, advanced glycation end-product; AL, acetylation; CarP, carbamylated protein; MAA, malondialdehyde-acetaldehyde adduct.

Following immunisation with MSA containing the different PTMs, we could readily detect (auto)antibodies in mice immunised with OVA, MSA-CarP, MSA-AGE, MSA-AL and MSA-MAA, as shown in figure 1A. Using this immunisation protocol, no antibodies were formed against MSA-Cit and MSA-NT. As expected, there was no response against unmodified MSA.

Next, we analysed avidity using an elution assay with sodium thiocyanate (NaSCN).^4^ We observed that the avidity of antibodies for MSA-CarP, MSA-AGE and MSA-AL was significantly lower than for the foreign antigen OVA (figure 1B). However, for MSA-MAA, a substantially higher avidity was observed in the same range as the avidity to OVA. Altogether, we observed that under these controlled conditions, most anti-PTM responses are of low avidity, with the exception of anti-MAA, suggesting that different types of B cell responses underlie these different anti-PTM responses. Anti-PTM avidity is unrelated to their concentration (online supplemental figure 1). We replicated these results in a small proof of concept experiment where we tested RA samples, obtained as anonymised left over samples from diagnostics, for the avidity of anti-tetanus toxoid (TT) antibodies as a recall antigen and the anti-PTM antibodies (figure 1C). We observed lower avidity for anti-Cit, anti-CarP and anti-AGE compared with anti-TT. Importantly, also in patients, the avidity of anti-MAA was higher and not different from anti-TT.

10.1136/rmdopen-2024-004131.supp2 Supplementary data

The higher avidity towards MAA may be explained by the fact that mice and humans at birth have germ-line encoded natural IgM anti-MAA antibodies.^6^ These existing B-cells could undergo different affinity maturation compared with de novo B cell responses. As observed before, we were unable to elicit ACPA responses in mice, under conditions where we could induce other anti-PTM antibodies. The avidity of the anti-Cit response observed in this small number of patients corresponds to the study of Yamada *et al* 3 and our earlier work.^4^

This research highlights that within the anti-PTM autoantibodies not only ACPA is of low avidity but also anti-CarP, anti-AL and anti-AGE. In contrast, anti-MAA antibodies have a clearly higher avidity, not different from recall antigens, underscoring different modes of B cell activation for different PTM.
