# Supplementary material for: Differences in avidity of anti-post-translationally modified protein antibodies in mouse models and rheumatoid arthritis patients: not one-size-fits-all
Source: RMD Open. 2024 May 24;10(2):e004131. doi: 10.1136/rmdopen-2024-004131 (PMC11129038; doi:10.1136/rmdopen-2024-004131)
Supplement: Supplementary data [file rmdopen-2024-004131supp001.pdf]

Methods

Animal experiments

All animal experiments were approved by the Ethical Committee for Animal Experimentation in Leiden, the Netherlands. Eight- to twelve-week-old male C57BL6/J mice were purchased from Charles River Laboratories. Mice were kept under regular light/dark cycles (12 h light:12 h dark) at room temperature with food available ad libitum. They were housed with four mice per cage. Mice were divided into eight different groups using random numbers generated using the standard =RAND() function in Microsoft Excel. Mice (n = 8 per group) were immunized four times subcutaneously with 20 µg of PTM-modified or unmodified mouse serum albumin or ovalbumin mixed 1:1 with Alhydrogel® adjuvant 2% (vac-alu-250, InvivoGen). Immunizations were performed at two-week intervals on day 0, day 14, day 28, and day 42. On day 49, mice were sacrificed, and blood was collected via heart puncture. Blood was collected in an Eppendorf tube and placed on ice. Serum was prepared by centrifugation using a tabletop centrifuge at 13,000 RPM for 10 minutes.

Detection of anti-PTM antibodies from mouse serum

We specifically use modified and non-modified FCS because it allows us to exclusively detect PTM specific antibodies. Utilizing mouse serum albumin (MSA) would not allow us to clearly differentiate between PTM-specific antibodies and those targeting MSA itself. Moreover, by employing both modified and unmodified FCS, we can subtract any reactivity against the unmodified FCS, thus isolating a highly specific signal for PTM antibodies. Detection of anti-PTM Ig antibodies by ELISA: Modified fetal calf serum (FCS) and non-modified FCS were coated at 10 µg/mL in 0.1 M carbonate–bicarbonate buffer pH 9.6 on Nunc Maxisorp plates (430341, Thermofisher) for one hour at 37°C. Between each step, plates were washed with Phosphate Buffered Saline (PBS)/0.05% Tween (P1379, Sigma). After washing, plates were blocked using PBS/1% Bovine Serum Albumin (BSA) for 1 hour at 37°C. Following washing, wells were incubated with serum at different dilutions in PBS/0.05% Tween/1% BSA (PTB) for Ovalbumin 1/10,000, Mouse serum albumin 1/50, carbamylation (CarP) 1/3,000, acetylation (AL) 1/3,000, malondialdehyde–acetaldehyde adducts (MAA) 1/3,000, advanced glycation end-products (AGE) 1/50, citrullination (Cit) 1/50, and nitration (NT) 1/50 in PTB, respectively.

For each PTM, a standard of a known concentration of mouse IgG antibody was taken along in serial dilutions on each plate. Serum was incubated for one hour at 37°C. Mouse Ig was detected using Goat anti-Mouse Ig-HRP (DAKO, P0447) diluted in PTB and incubated at 37°C for 1 hour. After the final wash, HRP enzyme activity was visualized using ABTS (A1888, Merck) with 0.05% H_2O_2 (107209, Merck), and absorbance at 415 nm was read using a microplate reader (Bio-Rad). Absorbance was transformed to arbitrary units per milliliter (AU/mL) using a corresponding standard line for each PTM. Background AU/mL of FCS was subtracted from the AU/mL signal on FCS-PTM to analyze specific anti-PTM reactivity. Negative outcomes were changed to 0.

Avidity of anti-PTM antibodies in mouse serum

Avidity of serum IgG was determined by an elution assay using sodium thiocyanate (NaSCN) according to a previous report by Suwannalai et al. with minor modifications. Antigen-coated plates, as described above, were incubated with optimally diluted serum for 1 h at 37°C. After washing three times with PBS/0.05% Tween, the plate wells were incubated with NaSCN at concentrations of 0 or 0.5 M for 15 min at 37°C. The plates were washed three times with PBS/0.05% Tween, and remaining bound IgG antibodies were detected using Goat anti-Mouse IgG γ chain Antibody, HRP conjugate, Species Adsorbed (Merck, AP503P). The avidity index was calculated as follows: Avidity Index = (The amount of residual antibodies bound to the antigen after elution with 0.5M NaSCN in AU/mL) divided by the amount of antibodies in the absence of NaSCN (0.0M) in AU/mL x 100 = %.

Human serum samples

Serum from nine anonymized Rheumatoid arthritis patients, whose samples were left over from routine diagnostics, were used as proof of concept. Seven of the patients were positive for tetanus toxoid antibodies, six for Cit and AGE, eight for CarP, and all nine for MAA. Only in one patient could AL antibodies be detected; this was left out of analyses.

Avidity of anti-PTM antibodies in human serum

Avidity of serum IgG was determined by an elution assay using sodium thiocyanate (NaSCN) according to a previous report by Suwannalai et al. with minor modifications. Antigen-coated plates, as described above, were incubated with optimally diluted serum for 1 h at 37°C. After washing three times with PBS/0.05% Tween, the plate wells were incubated with NaSCN at concentrations of 0 or 1 M for 15 min at 37°C. The plates were washed three times with PBS/0.05% Tween, and remaining bound antibodies were detected using Rabbit-anti-Human IgG-HRP (Dako, P0214). The avidity index was calculated as follows: Avidity Index = (The amount of residual antibodies bound to the antigen after elution with 1 M NaSCN in AU/mL) divided by the amount of antibodies in the absence of NaSCN (0.0M) in AU/mL x 100 = %.

Antibody levels do not correlate with avidity index

To study if the antibody levels presented in Figure 1A correlate with the avidity index shown in Figure 1B we correlated antibody levels to avidity. We observed no significant correlation between antibody levels and the avidity index (Supplementary Figure 1).

Supplementary Figure 1

Legend Supplementary Figure 1.

Supplementary Figure 1: Correlation between antibody levels and the avidity index. Across all four PTMs that elicit antibody responses, the antibody levels demonstrate no correlation with the avidity index. Statistics: not significant (ns), $P > 0.05$.

Statistics

To analyze the difference in avidity among antibodies, a multiple comparison analysis was performed using one-way ANOVA with Dunnett post hoc testing. Correlations between the antibody levels and the avidity index were tested with the Pearson Correlation Coefficients. All analyses were conducted with GraphPad-Prism 9.0.1 (GraphPad Software, La Jolla, CA).
