# Supplementary figures and images for: Differences in avidity of anti-post-translationally modified protein antibodies in mouse models and rheumatoid arthritis patients: not one-size-fits-all
Source: RMD Open. 2024 May 24;10(2):e004131. doi: 10.1136/rmdopen-2024-004131 (PMC11129038; doi:10.1136/rmdopen-2024-004131)

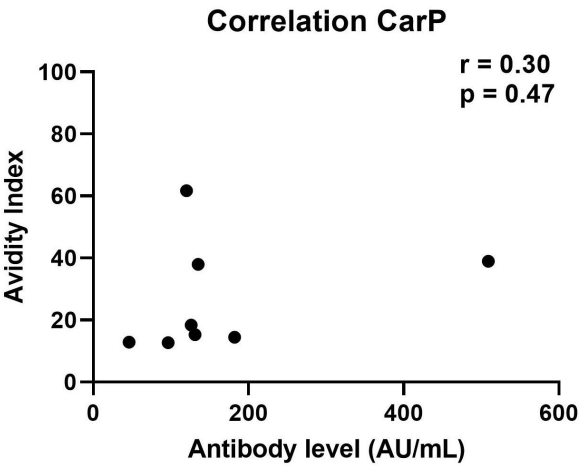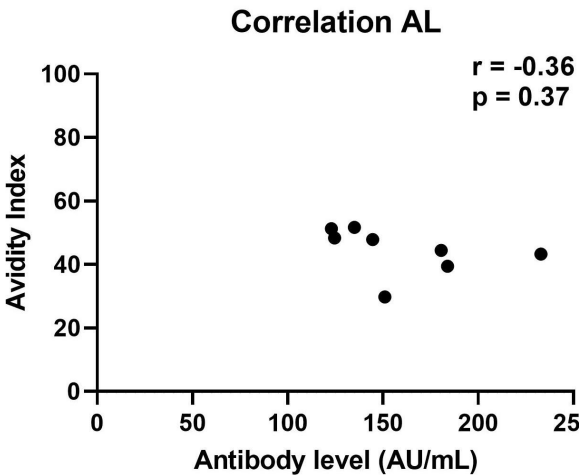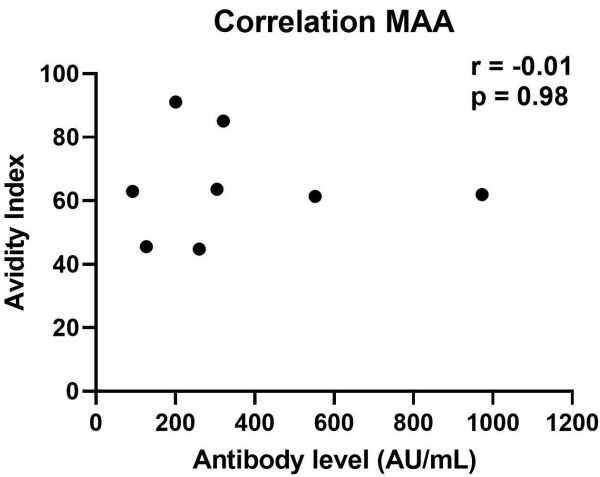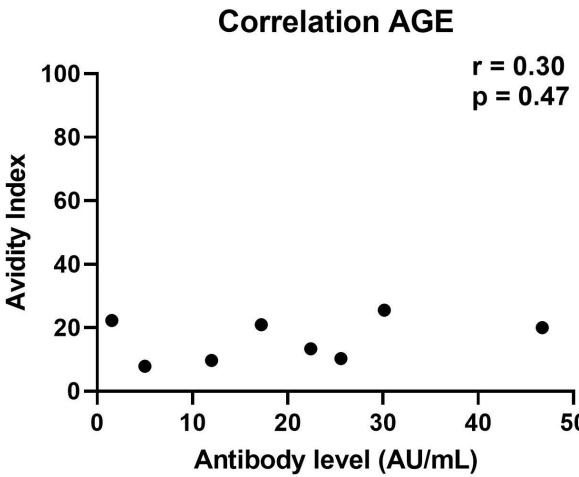

Supplement: Supplementary data [file rmdopen-2024-004131supp002.pdf]
